# Associations of birth weight, linear growth and relative weight gain throughout life with abdominal fat depots in adulthood: the 1982 Pelotas (Brazil) birth cohort study

**DOI:** 10.1038/ijo.2015.192

**Published:** 2015-10-13

**Authors:** G V Araújo de França, E De Lucia Rolfe, B L Horta, D P Gigante, J S Yudkin, K K Ong, C G Victora

**Affiliations:** 1 Post-graduate Program in Epidemiology, Federal University of Pelotas, Pelotas, Brazil; 2 Medical Research Council (MRC) Epidemiology Unit, Institute of Metabolic Science, University of Cambridge School of Clinical Medicine, Cambridge Biomedical Campus, Cambridge, UK; 3 University College London, London, UK

## Abstract

**Background:**

Several studies have reported on associations of size at birth and early growth with general and central obesity; however, few have examined the potential effects of birth weight and postnatal growth on separate abdominal fat compartments. We investigated the effects of size at birth, linear growth and relative weight gain from birth to adulthood on visceral (VFT) and subcutaneous abdominal (SAFT) fat thicknesses at age 30 years.

**Methods:**

A total of 2663 participants from the 1982 Pelotas (Brazil) birth cohort study had complete information on ultrasound measures of abdominal fat at age 30 years, and anthropometric measurements for at least five visits (0/2/4/23/30 years). We estimated weight and height *Z*-score changes, conditional relative weight gain and conditional height at several ages.

**Results:**

In both men and women, VFT and SAFT showed positive associations with conditional relative weight gain during all age periods beyond 2 years and birth, respectively (all *P*⩽0.01). Women born with intrauterine growth restriction (IUGR) had greater VFT than other women (difference=0.15 s.d., 95% CI: 0.01–0.29), and they showed a stronger positive influence of infant weight gain 0–2 years on VFT (IUGR: *β*=0.17 s.d., 95% CI: 0.05–0.29; non-IUGR: *β*=0.01 s.d., 95% CI: −0.04 to 0.06; *P*_interaction_=0.02). Stunting at 2 years was associated with lower SAFT but not VFT, and it modified the influence of weight gain 2–4 years on SAFT in both sexes (both *P*_interaction_<0.05).

**Conclusions:**

Our findings reinforce the advantages of being born with an appropriate birth weight, and the hazards of rapid postnatal gains in weight relative to linear growth, particularly after the critical window of the first 1000 days.

## Introduction

It has been hypothesised that physical and social exposures acting early in life may have long-term impact on obesity, also possibly affecting regional fat distribution.^1^ According to the ‘developmental origins of health and disease hypothesis', adverse conditions in early development, either *in utero* or in the early postnatal years or both, may lead to metabolic changes that increase the risk of disease later in life.^1, 2, 3^ Birth weight (BW) has been extensively used as proxy for intrauterine growth and positive associations with adult body mass index (BMI) have been consistently reported.^4, 5^ A recent meta-analysis also reported that BW shows consistent positive associations with other markers of adult adiposity, including waist and hip circumferences.^6^ Conversely, when adjustment is included for current BMI, the associations between BW and central adiposity usually become inverse, a phenomenon that has been attributed to positive associations with postnatal weight gain;^7^ nevertheless, this interpretation is largely unconfirmed due to the lack of postnatal growth data in most studies.^6^ Furthermore, such studies invariably analysed waist circumference as a crude estimate of visceral fat quantity.^6, 8^

In addition to the positive associations of BW and postnatal weight gain with later adiposity, foetal growth restriction or growth trajectories in the first 2 years of life are related to irreversible damage, such as shorter adult height and decreased offspring BW; however, studies on the associations of rapid weight or length gain in the first 2 years of life with later development of chronic disease are inconclusive.^9^ A recent review of studies in high-income countries reported strong positive associations between rapid growth during infancy and early childhood and adult obesity in high-income countries.^4^ In addition, findings from five low- and middle-income countries suggest that weight trajectories in the first 2 years of life are more strongly associated with adult lean mass than with fat mass, whereas the weight gain between 2 and 4 years (early childhood) is associated with both fat and lean masses.^10^ Previous results from the 1982 Pelotas (Brazil) birth cohort study have shown positive associations of waist circumference adjusted for hip circumference at age 23 years (considered as a proxy for visceral adipose tissue) with weight gain after 4 years in men and after 2 years in women.^11^ Nevertheless, studies on the associations of rapid weight gain with abdominal fat depots measured through imaging methods are scarce and restricted to high-income settings.^12, 13^

Several factors are involved in the growth process and physiological development, and adverse conditions may potentially modify the child's growth trajectory. An unfavourable intrauterine environment can prevent the foetus from attaining its growth potential and result in adaptive problems. This process – so-called ‘intrauterine growth restriction' (IUGR)^14^ – has been associated with perinatal and long-term increased health-related risk, and seems to be related to rapid postnatal growth and increased fat accumulation.^11, 15^ In addition, postnatal undernutrition or excessive weight gain in the 1 year of life, arising from IUGR or not, may influence fat accumulation and constitute protective or risk factors for later adverse outcomes.^16, 17^

In the present study, we aimed to: (i) test the hypotheses that higher weights and faster growth in childhood would increase abdominal fat in adults from a middle-income setting and (ii) investigate whether the above trajectory is modified by IUGR or nutritional status at age 2 years.

## Materials and methods

### Study design and participants

The 1982 Pelotas Birth Cohort Study has been previously described.^18, 19, 20^ Briefly, it recruited 5914 live births in 1982 to mothers living in the urban area of Pelotas, a city in Southern Brazil. The first follow-up in 1983 targeted only children born between January and April 1982; 1457 children were examined (~80% of the target sample). In 1984 and 1986, all urban households were visited, resulting in follow-up rates of 87% (*n*=4934) and 84% (*n*=4742), respectively. In 1997, 70 census tracts (27% of the total) were randomly selected and visited to locate 1076 cohort members (72% of the target). In 2000, 2250 males (78.9% of the target) were interviewed and had physical examinations at compulsory Army recruitment. In 2001, all households in the 70 census tracts selected in 1997 were revisited, locating 1031 women (69% of target). In 2004–2005, another household census was performed and 4297 cohort members (77% of target) were interviewed and examined. Most recently, in 2012–2013, 3711 cohort members attended a research clinic follow-up (68.2% of target). In the early phases, verbal informed consent was obtained from the mothers, whereas in recent phases, written consent was obtained. Study protocols were approved by the Ethical Committee of the Federal University of Pelotas.^19^

### Anthropometric data

Trained anthropometrists collected the measurements following standard protocols. Infants were weighed to the nearest 10 g using calibrated pediatric scales (Filizola, São Paulo, Brazil). Children were weighed to the nearest 0.1 kg using portable mechanical scales (CMS Weighing Equipment Ltd, London, UK). Adolescents and adults were weighed to the nearest 0.1 kg using calibrated electronic scales (TANITA BC-418 MA; Tanita, Tokyo, Japan).

Birth length was not recorded. Supine length and height was measured to the nearest 0.1 cm in 1984 and 1986, respectively, using boards manufactured locally according to international specifications (AHRTAG; Healthlink Worldwide, London, UK). From 1997 onwards, standing height was measured to the nearest 0.1cm using a full-length wall-mounted stadiometer (SECA 240; Seca, Birmingham, UK).

BW and childhood measurements were converted to age and sex-specific *Z*-scores by comparison with the 2006/2007 World Health Organization (WHO) growth standards.^21^ Age and sex-specific internal *Z*-scores were calculated for weight and height at ages 18/19, 23 and 30 years, excluding pregnant or post-partum women. We combined data from 2000 (males) and 2001 (females) as follow-up at age 18/19 years. IUGR was defined as BW for gestational age and sex below the 10th centile using the reference developed by Williams *et al.*^22^ Stunting (height *Z*-score below –2) and overweight (BMI *Z*-score above +2) at age 2 years were defined by 2006 WHO criteria.^21^

### Abdominal fat at age 30 years

Abdominal ultrasound was performed using a Toshiba Xario (Toshiba Medical Systems Corp., Tokyo, Japan) ultrasound machine with a 3.5-MHz convex probe following validated protocols.^23, 24^ Briefly, visceral fat thickness (VFT) was estimated by the distance between the peritoneum and the lumbar spine at the intersection between the xyphoid line and the waist circumference. Subcutaneous abdominal fat thickness (SAFT) was estimated at the same probe site by the distance between the posterior line of dermis and the outer bowel wall. Both distances were measured from static images taken at the end of a quiet expiration when applying minimal pressure. Women with known or probable pregnancy, or up to 3 months post-partum, were excluded from this assessment. The relative intra-observer technical error of measurement for the visceral thickness was 4.1% and 3.4% for subcutaneous fat thickness, whereas the relative inter-observer technical error of measurement was 3.1% for both measurements.

### Other variables

Data on *a priori* potential confounders were collected.^19, 20^ Family income in 1982 was calculated as the sum of the monthly incomes of all working persons living in the household, expressed in multiples of the minimum wage (⩽1/1.1–3/>3). Gestational age was calculated by mother's recalled last menstrual period. For 522 study members, gestational age was missing or invalid; for 507 of these, multiple imputed values (five imputations) were calculated using a regression model containing maternal age, skin colour, height, smoking during pregnancy, offspring sex and family income at birth.^25^ Maternal education was assessed as completed years of formal education, and was categorised into three groups (0–4/5–8/9+ years); maternal self-reported skin colour was categorised into two groups (white/non-white); maternal smoking during pregnancy was categorised into five groups (non-smokers/1–14 cigarettes during part of pregnancy/1–14 cigarettes during whole of pregnancy/15+ cigarettes during part of pregnancy/15+ cigarettes during whole of pregnancy). Maternal height was measured and pre-pregnancy weight was obtained from the antenatal care register or reported by the mother and used to calculate pre-pregnancy BMI. Missing BMI values were imputed using the same method as described for gestational age.^25^

### Statistical analysis

Statistical analyses were performed using Stata version 13 (StataCorp, College Station, TX, USA) and all analyses were stratified by sex. VFT and SAFT were transformed to normal distributions using logarithmic and square root functions, respectively. Both variables were posteriorly standardised to allow direct comparisons of the regression coefficients for these outcomes.

Growth was analysed separately by sex, in subjects with complete anthropometry and ultrasound data, excluding women who were pregnant or post-partum in 2000, 2004–2005 and 2012–2013. In all 2 men and 22 women had undergone abdominal plastic surgery, but when these individuals were excluded from the analyses the results remained unchanged (data not shown). Therefore, we present the results on the whole available sample.

Conditional growth models were performed by generating conditional weight or height measures that are uncorrelated with all previous measurements and therefore indicate the influence of recent growth rate (since the preceding time point) on the ultrasound outcome measure.^26, 27, 28^ Conditional relative weight gain was estimated by regressing the weight at each age on all previous weight and height measures, and also concurrent height. Conditional height was estimated by regressing the height at each age on all previous weight and height measures, but not concurrent weight.^26^

Multiple linear regression models tested the associations with VFT or SAFT and *Z*-scores for BW and attained weight and height at ages 1, 2, 4, 15, 18–19, 23 or 30 years; conditional relative weight gain at ages 2, 4, 23 or 30 years; and conditional relative height gain at ages 4 or 23 years. Additional analyses were carried out for shorter age ranges based on the subsamples measured at ages 1, 15 and 18/19 years. Models were adjusted for family income at birth, maternal education, maternal skin colour, maternal height, maternal BMI before the pregnancy, smoking in pregnancy and gestational age.

Beta (*β*) coefficients indicate the standardised difference in VFT or SAFT per +1 s.d. of the exposure. To aid interpretation, some coefficients were back transformed using the inverse function and expressed in centimetres (cm). Baseline models for changes in weight or length/height *Z*-score were adjusted for weight or length/height *Z*-score at the beginning of the period.

Comparison with previous reports, unconditional growth models were also performed. For these, changes in weight or height *Z*-scores during each age period were calculated by subtracting the earlier from the later *Z*-score, and baseline regression models adjusted for weight or height at baseline.

Associations with, and the potential modifying role of, IUGR, stunting and overweight at age 2 years were tested. *β*-coefficients for the associations between weight gain and VFT or SAFT were calculated separately within those subgroups, as well as the interaction *P*-value. These models were adjusted for all potential confounders as above.

## Results

Valid data on VFT and SAFT at age 30 years were collected on 1724 men and 1769 women. The main analyses were performed on 1363 men and 1300 women with anthropometric measurements at four follow-up visits (at ages 2/4/23/30 years). This sample was similar to the original cohort (*n*=5914) with regard to sex, maternal skin colour and education, and prevalence of IUGR and overweight at 2 years; however, the selected sample had slightly higher family income at birth (*P*=0.004) and lower prevalence of low BW (*P*<0.001; Supplementary Table 1S).

Anthropometric measurements are summarised in Table 1. Men had higher median VFT at 30 years than women (7.0 cm vs 4.9 cm; *P*<0.001), but lower SAFT (1.9 cm vs 2.6 cm; *P*<0.001); both differences persisted after adjustment for BMI (all *P*<0.001; not shown).

Family income at birth and maternal education were inversely associated with VFT in females and positively related to SAFT in males. Regarding maternal skin colour, men born to non-white mothers had greater VFT in comparison with those born to white mothers; conversely, women born to non-white mothers showed, on average, lower SAFT than those whose mothers were white (*P*<0.001). Maternal height showed a strong positive association with SAFT, but only among males; in addition, we found weak but significant positive associations between maternal BMI before pregnancy and both fat compartments in both sexes. Smoking during pregnancy was inversely related to both VFT and SAFT, but only in women (Supplementary Table 2S).

### Attained body size and adult abdominal fat

Table 2 shows the adjusted associations for VFT and SAFT with attained weight and height at each age (unadjusted associations are shown in Supplementary Table 3S). BW showed no linear associations with VFT or SAFT at 30 years. We also tested for possible nonlinear associations between BW and VAT or SCAT by including in the models a BW-squared variable, and found no evidence to support a nonlinear relationship with BW (data not shown).

VFT was positively associated with attained weight from age 4 onwards in men, and only from age 15 in women. In contrast, inverse associations between VFT and attained height were found at most ages in women. SAFT was positively associated with attained weight and height at all ages in men and women, except at birth.

### Conditional growth and adult abdominal fat

Conditional relative weight gain from age 2 years onwards, but not conditional height, was positively associated with VFT (Table 3 and Supplementary Table 4S). SAFT was positively associated with conditional relative weight gain in all periods, with stronger associations seen in adolescence and early adulthood.

Generally similar associations were seen in unconditioned growth models (Supplementary Table 5S), but with some additional associations with height. In men, height gain between 4 and 15 years was positively associated with VFT, but, conversely, in women height gain between 4 and 23 years was inversely associated with VFT. In men and women, height gain between 2 and 4 years was positively associated with SAFT. Conversely, in women only, height gain between 4 and 23 years was inversely associated with SAFT.

### IUGR and stunting or overweight at age 2 years

Women born with IUGR had higher adult VFT than other women (mean difference: 0.7 cm, 95% CI: 0.6–0.8, *P*=0.01), but no difference in SAFT (*P*=0.33). By contrast, men born with IUGR had lower adult SAFT than other men (0.2 cm, 95% CI: 0.1, 0.3 cm, *P*<0.001), but no difference in VFT (*P*=0.35). Regarding nutritional status at age 2 years, men and women who were stunted had lower adult SAFT than other individuals (mean difference in men: 0.3 cm, 95% CI: 0.2, 0.4 cm, *P*<0.001; in women: 0.2 cm, 95% CI: 0.1, 0.3 cm, *P*=0.03), whereas those who were overweight at age 2 years had higher SAFT (mean difference in men: 0.4 cm, 95% CI: 0.2, 0.6 cm, *P*<0.001; in women: 0.5 cm, 95% CI: 0.2, 0.8 cm, *P*<0.001; Supplementary Table 6S).

We conducted interaction analyses in order to test if IUGR, stunting and overweight at 2 years modified the associations between early conditional weight gain and adult VFT or SAFT. In fully adjusted models, 3 out of 16 interaction tests were significant at the 5% level (Table 4). Early conditional weight gain (between 0 and 2 years) showed a stronger positive association with VFT in IUGR girls (*β*=0.17; *P*=0.004) than in non-IUGR girls (*β*=0.01; *P*=0.70; *P*-interaction=0.02). Furthermore, conditional weight gain (between 2 and 4 years) showed stronger positive associations with SAFT in non-stunted men and women than in stunted individuals (*P*-interaction in men=0.005, in women=0.02).

## Discussion

The findings from this large, long-running, middle-income country birth cohort study suggest differences in the relative contributions of childhood weight gain and linear growth to the two abdominal fat compartments, VFT and SAFT, in adults. In both men and women, higher relative conditional weight gain during most ages from infancy to adulthood was associated with greater VAT and SAFT. Moreover, the associations of weight gain early in life with abdominal fat appeared to be modified by IUGR (girls only) and stunting at age 2 years. In women, shorter attained height from infancy to adulthood was associated with higher VFT, whereas height gains were positively associated with SAFT.

Growth patterns throughout life have been associated with obesity risk in adulthood; the most consistent associations are with rapid growth during infancy and early childhood and with early BMI rebound.^4, 26^ Nevertheless, few studies have investigated such growth patterns in relation to the abdominal fat mass.^12, 13^ Several statistical approaches have been described to identify growth trajectories or critical age periods related to later disease risk.^29^ In our study, the conditional growth measures represent the acceleration or deceleration in growth from an individual's previous trajectory.

Our results suggest that rapid weight gain (independent of changes in height) promotes the accumulation of both VAT and SAFT, in both sexes; no critical windows were obvious –associations were apparent with weight gain at all ages from 2 years onwards for VAT, and at all ages from birth onwards for SAFT. However, the *β* coefficients for these associations strengthened substantially with age, which is consistent with the progressive increase in adiposity that begins at the BMI rebound period (at 3–7 years). A one s.d. increase in conditional relative weight gain from 2 to 4 years predicted a small increment (0.2 cm) in VFT in adulthood in both sexes, whereas the same increase in weight gain from 4 to 23 years predicted a more substantial increment (0.9 cm on average). We should point out that a one s.d. increase in conditional relative weight gain represents a large change in weight trajectory, and the estimated coefficients on adult VFT are small given the wide range in this measurement (2–16 cm).

We found no association between BW and adult abdominal fat mass, a finding that is consistent with the few studies, all set in high-income countries, that used direct imaging methods.^6, 13, 23, 30^ However, when we considered BW as a categorical variable, IUGR in women was associated with higher adult VFT, and a positive association between infant weight gain (0 –2 years) and VFT was seen in IUGR but not in non-IUGR women. Nevertheless, we found no evidence of interaction between IUGR and later rapid weight gain (after 2 years of age) on VFT or SAFT. These findings may seem contrary to previous studies that suggested a protective role of early rapid weight gain in the first 2 years of life (so-called early catch-up) against overall adiposity, in comparison with adverse consequences of later rapid weight gain (at 2–4 years of age).^31^ However, our current findings with these specific abdominal fat measures, in particular VFT, may have stronger relevance to metabolic disease risk.^8^ Furthermore, the accumulation of visceral and subcutaneous adipose tissues may involve different physiological mechanisms,^32, 33^ and in light of the substantial recent increase in the prevalence of obesity in our cohort (from 8.5% in 2004–2005 to 23.6% in 2012–2013), it may prove to be more beneficial to gain subcutaneous abdominal fat instead of visceral fat.

Previous studies have reported that linear growth retardation in early childhood is associated with short stature and less lean mass in adulthood; however, such studies have not specifically measured abdominal fat.^9, 28, 34^ Our results suggest that stunting at 2 years may limit the accumulation of SAFT but not VFT in both sexes; an ongoing preference to accumulate VFT rather than SAFT might be deleterious to metabolic health. In support of this idea, we observed that women with shorter height had more VFT, but unfortunately our lack of data on linear measurements at birth meant we were unable to distinguish between antenatal and early postnatal growth retardation.

We acknowledge the other limitations of our study. First, cohort members who attended the latest assessment tended to be wealthier and less likely to have low BW; these differences can be partly attributed to survival bias, as deaths in early life were more common among the poor and low BW children.^19^ The lower prevalence of low BW in subjects measured at 30 years of age is largely due to the fact that low BW infants in the original cohort were more likely to die and, therefore, less likely to be available for the follow-up at 30 years of age. In addition, we cannot rule out the possibility that selective survival of a subgroup of low BW newborns (e.g., those who presented faster weight gain in early life) may partly explain the association between IUGR and VFT in women. Second, many participants had missing values for gestational age and maternal pre-gestational weight and, therefore, we used a multiple imputation approach. Third, our main results are restricted to subjects with complete information on growth measurements at birth and at least five follow-up examinations. Further measurements at ages 1, 15 and 18/19 years were available in only small subsamples, and therefore we had limited ability to determine associations with specific age periods of growth, such as the BMI rebound and weight gain during adolescence. Finally, we relied on ultrasound measurements of VAT and SAFT as proxies for these abdominal fat masses; however, previous studies assessing the validity of the same protocol reported strong correlations with magnetic resonance imaging estimates of abdominal fat in a variety of settings and populations.^24, 35, 36^ A strict quality control process was carried out and we could identify consistent sex differences in the distribution of these abdominal fat compartments compared with previous reports using other imaging methods.^23, 37, 38, 39^

We therefore consider that our study represents an advance on previous knowledge regarding the early determinants of abdominal obesity in our cohort, by assessing separately the visceral and subcutaneous abdominal fat compartments. Moreover, we applied complementary methods to analyse growth trajectories associated with these outcomes, including relative growth and weight gain, which allows assessment of age-specific conditional measures independent of all previous weight and height measures, takes into account the strong correlation between repeated measurements over time and avoids the ‘reversal paradox'.^26, 40^

In conclusion, rapid weight gain in different age periods, but particularly after the age of 2 years, was associated with greater adult abdominal fat. In addition to rapid weight gain, markers of growth restraint, IUGR and short stature, appeared to confer greater susceptibility to accumulating visceral fat in women and influences abdominal subcutaneous fat in both sexes. Previous analyses from this cohort have been shown to be consistent with those in other birth cohorts set in lower- and middle-income countries. Thus, we consider that our findings point to metabolically adverse combinations of early growth restraint and rapid weight gain in populations in these settings. In terms of policy implications, our findings reinforce the advantages of being born with an appropriate BW, and the hazards of rapid postnatal gains in weight relative to linear growth, particularly after the critical window of the first 1000 days.

## Figures and Tables

**Table 1:** Anthropometric measurements and indicators from birth to age 30 years in males (*n*=1363) and females (*n*=1300)

*Measure/age*	*Males*			*Females*			P-*value*
	N	*Mean*	*s.d.*	N	*Mean*	*s.d.*	
*Weight (kg)*							
Birth	1363	3.30	0.52	1300	3.17	0.51	<0.001^a^
1 year	356	9.73	1.20	376	9.13	1.24	<0.001^a^
2 years	1363	11.38	1.58	1300	10.85	1.56	<0.001^a^
4 years	1363	15.79	2.20	1300	15.30	2.34	<0.001^b^
15 years	315	57.91	12.26	308	54.84	10.76	<0.001^b^
18/19 years^c^	1279	67.84	12.72	539	58.09	12.95	<0.001^a^
23 years^d^	1363	72.62	13.90	1300	60.52	12.35	<0.001^b^
30 years	1363	82.61	17.03	1300	69.79	16.05	<0.001^a^

*Height (cm)*							
Birth							
1 year	356	73.73	3.08	376	72.44	3.44	<0.001^b^
2 years	1363	81.34	4.77	1300	80.26	4.86	<0.001^a^
4 years	1363	98.02	5.03	1300	97.02	5.07	<0.001^a^
15 years	315	166.44	7.82	308	159.44	6.60	<0.001^b^
18/19 years^c^	1280	173.52	6.75	539	160.46	6.16	<0.001^b^
23 years^d^	1363	173.84	6.83	1300	160.97	6.15	<0.001^b^
30 years	1363	174.44	6.86	1300	161.43	6.11	<0.001^b^

*Weight (*Z-*score)*							
Birth	1363	–0.15	1.11	1300	–0.20	1.16	0.20^a^
1 year	356	0.19	1.09	376	0.24	1.07	0.90^a^
2 years	1363	0.09	1.08	1300	0.15	1.01	0.12^b^
4 years	1363	0.06	1.03	1300	−0.04	1.03	0.005^a^
15 years	315	0.01	0.97	308	0.00	1.03	0.82^a^
18/19 years^c^	1279	0.04	1.00	539	0.00	1.04	0.68^a^
23 years^d^	1363	0.05	1.00	1300	0.00	0.98	0.41^a^
30 years	1363	0.02	1.01	1300	0.02	1.00	0.99^a^

*Height (*Z-*score)*							
Birth							
1 year	356	−0.45	1.17	376	−0.24	1.17	0.01^a^
2 years	1363	−0.72	1.22	1300	−0.60	1.17	0.004^a^
4 years	1363	−0.60	1.11	1300	−0.67	1.09	0.27^a^
15 years	315	0.06	0.97	308	0.00	1.02	0.34^a^
18/19 years^c^	1280	0.02	1.00	539	0.01	0.99	0.92^a^
23 years^d^	1363	0.02	0.99	1300	0.04	0.98	0.83^a^
30 years	1363	0.00	1.00	1300	0.01	0.99	0.97^a^

Note: the weights and heights in childhood were transformed to *Z*-scores of weight/height for age and sex, using the 2006 WHO growth standards. Internal weight and height *Z*-scores were calculated using sex-specific distributions from the whole cohort for the ages 23 and 30 years excluding pregnant or post-partum women in the last two follow-ups from calculations to generate *Z*-scores.

a *t*-test.

b Kruskal–Wallis equality-of-populations rank test.

c Excluding 27 pregnant women in 2000.

d Excluding 20 pregnant and 8 post-partum women in 2004–2005.

**Table 2:** Attained weight or height from birth to 30 years associated with adult visceral or subcutaneous abdominal fat by sex

*Outcomes/age*	*Weight (Z-score)*					*Height (Z-score)*				
	N	β^a^	*95% CI*		P	N	β^a^	*95% CI*		P
*Visceral fat thickness (s.d. ln cm)*										
** **Males										
** **Birth	1626	0.03	−0.01	0.07	0.14					
** **1 year	416	0.07	−0.01	0.15	0.07	416	0.01	−0.07	0.08	0.85
** **2 years	1484	0.03	−0.01	0.07	0.14	1484	0.02	−0.02	0.06	0.39
** **4 years	**1458**	**0.07**	**0.02**	**0.11**	**0.003**	1457	0.04	−0.01	0.08	0.10
** **15 years	**364**	**0.18**	**0.09**	**0.28**	**<0.001**	**364**	**0.14**	**0.04**	**0.24**	**0.006**
** **18/19 years	**1490**	**0.27**	**0.23**	**0.31**	**<0.001**	1491	0	−0.05	0.05	0.97
** **23 years	**1508**	**0.38**	**0.34**	**0.42**	**<0.001**	1509	0	−0.05	0.05	0.92
** **30 years	**1624**	**0.53**	**0.5**	**0.56**	**<0.001**	1624	−0.01	−0.06	0.04	0.66
** **Females										
** **Birth	1612	−0.03	−0.07	0.02	0.22					
** **1 year	451	−0.05	−0.14	0.04	0.26	450	−0.08	−0.17	0.01	0.07
** **2 years	1485	0	−0.05	0.05	0.94	**1485**	**−0.05**	**−0.09**	**0**	**0.04**
** **4 years	1447	0.02	−0.03	0.07	0.38	1447	−0.02	−0.07	0.03	0.38
** **15 years	**351**	**0.2**	**0.1**	**0.3**	**<0.001**	351	−0.03	−0.14	0.09	0.64
** **18/19 years^b^	**623**	**0.2**	**0.12**	**0.27**	**<0.001**	**623**	**−0.09**	**−0.18**	**−0.01**	**0.04**
** **23 years^c^	**1387**	**0.38**	**0.34**	**0.43**	**<0.001**	**1486**	**−0.06**	**−0.12**	**0**	**0.04**
** **30 years	**1609**	**0.55**	**0.51**	**0.59**	**<0.001**	**1609**	**−0.07**	**−0.13**	**−0.02**	**0.007**

*Subcutaneous abdominal fat thickness (s.d. sqrt cm)*										
** **Males										
** **Birth	1626	0.02	−0.02	0.07	0.3					
** **1 year	**416**	**0.1**	**0.02**	**0.18**	**0.02**	416	0.03	−0.06	0.11	0.51
** **2 years	**1484**	**0.15**	**0.11**	**0.2**	**<0.001**	**1484**	**0.12**	**0.07**	**0.16**	**<0.001**
** **4 years	**1458**	**0.27**	**0.22**	**0.32**	**<0.001**	**1457**	**0.18**	**0.13**	**0.23**	**<0.001**
** **15 years	**364**	**0.45**	**0.37**	**0.54**	**<0.001**	**364**	**0.15**	**0.06**	**0.25**	**0.002**
** **18/19 years	**1490**	**0.53**	**0.48**	**0.57**	**<0.001**	**1491**	**0.13**	**0.07**	**0.18**	**<0.001**
** **23 years	**1508**	**0.59**	**0.55**	**0.63**	**<0.001**	**1509**	**0.11**	**0.06**	**0.17**	**<0.001**
** **30 years	**1624**	**0.69**	**0.66**	**0.73**	**<0.001**	**1624**	**0.11**	**0.05**	**0.16**	**<0.001**
** **Females										
** **Birth	1612	0.01	−0.04	0.05	0.79					
** **1 year	451	0.09	0	0.18	0.05	451	0.04	−0.04	0.13	0.33
** **2 years	**1485**	**0.16**	**0.1**	**0.21**	**<0.001**	**1485**	**0.06**	**0.01**	**0.1**	**0.02**
** **4 years	**1447**	**0.24**	**0.19**	**0.29**	**<0.001**	**1447**	**0.11**	**0.05**	**0.16**	**<0.001**
** **15 years	**351**	**0.58**	**0.49**	**0.66**	**<0.001**	**351**	**0.16**	**0.04**	**0.28**	**0.008**
** **18/19 years^b^	**623**	**0.47**	**0.4**	**0.54**	**<0.001**	623	0.08	−0.01	0.17	0.09
** **23 years^c^	**1387**	**0.63**	**0.59**	**0.68**	**<0.001**	1486	0.02	−0.04	0.08	0.52
** **30 years	**1609**	**0.76**	**0.73**	**0.79**	**<0.001**	1609	−0.01	−0.06	0.05	0.77

a Coefficients are from multiple linear regression and represent difference in the outcome per one s.d. increase in the exposure, adjusted for confounders measured at the time of birth: family income at birth, maternal education, maternal skin colour, maternal height, maternal BMI before the pregnancy (imputed), smoking in pregnancy and gestational age (imputed).

b Excluding 27 pregnant women in 2000.

c Excluding 20 pregnant and 8 post-partum women in 2004–2005. Bold values signifies *P*<0.05.

**Table 3:** Conditional growth in weight or height associated with adult visceral or subcutaneous abdominal fat, by sex

*Sex/age*	*Conditional relative weight gain*				*Conditional height*			
	β^a^	*95% CI*		P-*value*	β^a^	*95% CI*		P-*value*
*Visceral fat thickness (s.d. ln cm)*								
** **Males		(N=1333)				(N=1333)		
** **0–2 years	0.02	−0.02	0.07	0.31				
** **2–4 years	**0.07**	**0.02**	**0.11**	**0.005**	0.02	−0.03	0.06	0.41
** **4–23 years	**0.38**	**0.34**	**0.42**	**<0.001**	−0.03	−0.08	0.01	0.18
** **23–30 years	**0.41**	**0.37**	**0.45**	**<0.001**				
** **Females^b^^,^^c^		(N=1276)				(N=1276)		
** **0–2 years	0.04	−0.01	0.08	0.16				
** **2–4 years	**0.07**	**0.02**	**0.12**	**0.01**	0.04	−0.01	0.08	0.15
** **4–23 years	**0.39**	**0.35**	**0.44**	**<0.001**	−0.04	−0.09	0.01	0.14
** **23–30 years	**0.42**	**0.37**	**0.47**	**<0.001**				

*Subcutaneous abdominal fat thickness (s.d. sqrt cm)*								
** **Males		(N=1333)				(N=1333)		
** **0–2 years	**0.10**	**0.05**	**0.15**	**<0.001**				
** **2–4 years	**0.22**	**0.16**	**0.27**	**<0.001**	**0.06**	**0.01**	**0.11**	**0.01**
** **4–23 years	**0.48**	**0.44**	**0.52**	**<0.001**	0.00	−0.05	0.05	0.94
** **23–30 years	**0.38**	**0.34**	**0.43**	**<0.001**				
** **Females^b^^,^^c^		(N=1276)				(N=1276)		
** **0–2 years	**0.14**	**0.09**	**0.19**	**<0.001**				
** **2–4 years	**0.23**	**0.17**	**0.28**	**<0.001**	0.03	−0.03	0.08	0.34
** **4–23 years	**0.57**	**0.53**	**0.62**	**<0.001**	−0.04	−0.1	0.01	0.14
** **23–30 years	**0.43**	**0.38**	**0.48**	**<0.001**				

a Adjusted for confounders measured at the time of birth: family income at birth, maternal education, maternal skin colour, maternal height, maternal BMI before the pregnancy (imputed), smoking in pregnancy and gestational age (imputed).

b Excluding 27 pregnant women in 2000.

c Excluding 20 pregnant and 8 post-partum women in 2004–2005.

Bold values signifies *P*<0.05.

**Table 4:** Coefficients from multiple linear regression relating ultrasound measurements of abdominal fat at age 30 years to conditional weight scores from birth to 4 years stratified by sex, intrauterine growth restriction, and stunting and overweight at age 2 years.

*Conditional relative weight gain*	*Visceral fat thickness (s.d. ln cm)*									*Subcutaneous abdominal fat thickness (s.d. sqrt cm)*								
	β	*95% CI*		P-*value*	β	*95% CI*		P-*value*	*Interaction* P-*value*	β	*95% CI*		P-*value*	β	*95% CI*		P-*value*	*Interaction* P-*value*
*Intrauterine growth restriction*																		
	Non-IUGR				IUGR					Non-IUGR				IUGR				
* *0–2 years																		
Males	0.02	−0.03	0.07	0.54	0.07	−0.03	0.16	0.16	0.53	0.08	0.02	0.14	0.007	0.20	0.08	0.31	0.001	0.10
Females	**0.01**	−**0.04**	**0.06**	**0.70**	**0.17**	**0.05**	**0.29**	**0.004**	**0.02**	0.15	0.10	0.21	<0.001	0.05	−0.09	0.19	0.47	0.19
2−4 years																		
Males	0.07	0.02	0.13	0.004	0.01	−0.10	0.12	0.84	0.29	0.22	0.16	0.28	<0.001	0.20	0.06	0.33	0.005	0.62
Females	0.06	0.00	0.12	0.04	0.15	0.04	0.26	0.01	0.22	0.23	0.17	0.28	<0.001	0.23	0.11	0.36	<0.001	0.76
				
*Stunting at 2 years*																		
	Non−stunted at 2 years				Stunted at 2 years					Non-stunted at 2 years				Stunted at 2 years				
2–4 years																		
Males	0.07	0.02	0.12	0.01	0.04	−0.07	0.15	0.47	0.47	**0.25**	**0.20**	**0.31**	**<0.001**	**0.08**	−**0.05**	**0.20**	**0.22**	**0.005**
Females	0.09	0.03	0.14	0.001	0.00	−0.15	0.14	0.95	0.16	**0.25**	**0.20**	**0.31**	**<0.001**	**0.07**	−**0.07**	**0.22**	**0.32**	**0.02**
				
*Overweight at 2 years*																		
	Non-overweight at 2 years				Overweight at 2 years					Non-overweight at 2 years				Overweight at 2 years				
* *2–4 years																		
Males	0.07	0.02	0.12	0.01	0.07	−0.07	0.22	0.33	0.64	0.21	0.16	0.27	<0.001	0.23	0.07	0.39	0.005	0.97
Females	0.08	0.02	0.13	0.004	0.08	−0.07	0.22	0.29	0.60	0.22	0.16	0.28	<0.001	0.23	0.09	0.37	0.002	0.80

All analyses also adjusted for confounders measured at the time of birth: family income at birth, maternal education, maternal skin colour, maternal height, maternal BMI before pregnancy (imputed) and smoking in pregnancy. Stunting and overweight in 1984 are also adjusted for birth weight and gestational age (imputed). Bold values signifies interaction *P*-value <0.05.

## Supplementary Material

Supplementary Table 1S

Supplementary Table 2S

Supplementary Table 3S

Supplementary Table 4S

Supplementary Table 5S

Supplementary Table 6S
