# Supplementary material for: Associations of birth weight, linear growth and relative weight gain throughout life with abdominal fat depots in adulthood: the 1982 Pelotas (Brazil) birth cohort study
Source: Int J Obes (Lond). 2015 Oct 13;40(1):14–21. doi: 10.1038/ijo.2015.192 (PMC4722236; doi:10.1038/ijo.2015.192)
Supplement: Supplementary Table 1S [file ijo2015192x1.docx]

**Table 1S.** Comparison of Demographic and Socioeconomic Characteristic at Birth and at 2y Between the Original Cohort and the Selected Sample*

| Variables | Original cohort | |  | Selected sample ^a,b^ | | p-value** |
| --- | --- | --- | --- | --- | --- | --- |
|  | N | % |  | N | % |  |
| Sex |  |  |  |  |  |  |
| Male | 3,037 | 51.4 |  | 1,363 | 51.2 | 0.89 |
| Female | 2,876 | 48.6 |  | 1,300 | 48.8 |  |
| Maternal skin colour |  |  |  |  |  |  |
| White | 4,851 | 82.1 |  | 2,187 | 82.1 | 0.98 |
| Black/mixed | 1,060 | 17.9 |  | 476 | 17.9 |  |
| Family income at birth (minimum wages) |  |  |  |  |  |  |
| ≤ 1 | 1,288 | 21.9 |  | 499 | 18.8 | **0.004** |
| 1.1 - 3 | 2,789 | 47.4 |  | 1,328 | 50.1 |  |
| >3 | 1,808 | 30.7 |  | 825 | 31.1 |  |
| Maternal education (years) |  |  |  |  |  |  |
| 0-4 | 1,960 | 33.2 |  | 847 | 31.9 | 0.31 |
| 5-8 | 2,454 | 41.5 |  | 1,150 | 43.3 |  |
| 9+ | 1,493 | 25.3 |  | 662 | 24.9 |  |
| Birthweight (g) |  |  |  |  |  |  |
| <2500 | 534 | 9.0 |  | 181 | 6.8 | **<0.001** |
| ≥2500 | 5,375 | 91.0 |  | 2,482 | 93.2 |  |
| IUGR |  |  |  |  |  |  |
| No | 3,977 | 85.2 |  | 2,231 | 84.2 | 0.29 |
| Yes | 692 | 14.8 |  | 417 | 15.8 |  |
| Stunting at 2y (HAZ<-2SD) |  |  |  |  |  |  |
| No | 4,243 | 86.0 |  | 2,328 | 87.4 | 0.09 |
| Yes | 690 | 14.0 |  | 335 | 12.6 |  |
| Overweight at 2y (BAZ>+2SD) |  |  |  |  |  |  |
| No | 4,511 | 91.5 |  | 2,432 | 91.3 | 0.78 |
| Yes | 419 | 8.5 |  | 231 | 8.7 |  |
| **Overall** | **5,914** | **-** |  | **2,663** | **45.1** |  |

Abbreviation: IUGR, intrauterine growth restriction.

*Participants with information on anthropometric variables at 0/2/4/23/30y and ultrasound measurements in 2012-13.

** Fisher’s exact test

^a^Excluding 27 pregnant women in 2000.

^b^Excluding 20 pregnant and 8 post-partum women in 2004–2005.
