# Supplementary material for: Associations of birth weight, linear growth and relative weight gain throughout life with abdominal fat depots in adulthood: the 1982 Pelotas (Brazil) birth cohort study
Source: Int J Obes (Lond). 2015 Oct 13;40(1):14–21. doi: 10.1038/ijo.2015.192 (PMC4722236; doi:10.1038/ijo.2015.192)
Supplement: Supplementary Table 2S [file ijo2015192x2.docx]

**Table 2S.** Associations between characteristics measured at the time of birth and adult visceral or subcutaneous abdominal fat, by sex.

| Variables | Visceral fat thickness (SD ln cm) | | | |  | Subcutaneous abdominal fat thickness (SD sqrt cm) | | | |
| --- | --- | --- | --- | --- | --- | --- | --- | --- | --- |
|  | **β** | **95%CI** | | **p-value** |  | **β** | **95%CI** | | **p-value** |
| Males |  |  |  |  |  |  |  |  |  |
| Family income at birth (minimum wages) |  |  |  |  |  |  |  |  |  |
| 1 or less | Ref |  |  | 0.45 |  | Ref |  |  | **<0.001** |
| 1.1 to 3 | 0.07 | -0.04 | 0.19 |  |  | 0.23 | 0.10 | 0.37 |  |
| >3 | 0.05 | -0.07 | 0.18 |  |  | 0.44 | 0.29 | 0.58 |  |
| Maternal education (years) |  |  |  |  |  |  |  |  |  |
| <4 | Ref |  |  | 0.23 |  | Ref |  |  | **<0.001** |
| 5 to 8 | 0.07 | -0.03 | 0.17 |  |  | 0.20 | 0.08 | 0.31 |  |
| 9+ | -0.01 | -0.13 | 0.10 |  |  | 0.28 | 0.15 | 0.42 |  |
| Maternal skin color |  |  |  |  |  |  |  |  |  |
| White | Ref |  |  | 0.08 |  | Ref |  |  | **<0.001** |
| Non-white | -0.10 | -0.21 | 0.01 |  |  | -0.23 | -0.36 | -0.11 |  |
| Maternal height (cm) | 0.13 | -0.56 | 0.82 | 0.71 |  | 1.72 | 0.91 | 2.53 | **<0.001** |
| Maternal BMI before pregnancy (kg/m^2^) | 0.02 | 0.00 | 0.03 | **0.01** |  | 0.05 | 0.04 | 0.06 | **<0.001** |
| Smoking in pregnancy |  |  |  |  |  |  |  |  |  |
| No | Ref |  |  | 0.34 |  | Ref |  |  | 0.91 |
| Yes | -0.04 | -0.13 | 0.05 |  |  | 0.01 | -0.10 | 0.11 |  |
| Females |  |  |  |  |  |  |  |  |  |
| Family income at birth (minimum wages) |  |  |  |  |  |  |  |  |  |
| 1 or less | Ref |  |  | **<0.001** |  | Ref |  |  | 0.09 |
| 1.1 to 3 | -0.06 | -0.20 | 0.07 |  |  | 0.08 | -0.06 | 0.22 |  |
| >3 | -0.32 | -0.46 | -0.17 |  |  | -0.05 | -0.21 | 0.10 |  |
| Maternal education (years) |  |  |  |  |  |  |  |  |  |
| <4 | Ref |  |  | **<0.001** |  | Ref |  |  | 0.06 |
| 5 to 8 | -0.05 | -0.17 | 0.06 |  |  | -0.01 | -0.13 | 0.12 |  |
| 9+ | -0.38 | -0.51 | -0.25 |  |  | -0.15 | -0.29 | -0.01 |  |
| Maternal skin color |  |  |  |  |  |  |  |  |  |
| White | Ref |  |  | **0.001** |  | Ref |  |  | 0.37 |
| Non-white | 0.22 | 0.09 | 0.35 |  |  | 0.06 | -0.08 | 0.20 |  |
| Maternal height (cm) | -0.62 | -1.48 | 0.24 | 0.16 |  | 0.76 | -0.16 | 1.67 | 0.10 |
| Maternal BMI before pregnancy (kg/m^2^) | 0.02 | 0.01 | 0.04 | **<0.001** |  | 0.05 | 0.03 | 0.06 | **<0.001** |
| Smoking in pregnancy |  |  |  |  |  |  |  |  |  |
| No | Ref |  |  | **0.04** |  | Ref |  |  | **0.03** |
| Yes | -0.11 | -0.22 | -0.01 |  |  | -0.12 | -0.23 | -0.01 |  |
