# Supplementary material for: Associations of birth weight, linear growth and relative weight gain throughout life with abdominal fat depots in adulthood: the 1982 Pelotas (Brazil) birth cohort study
Source: Int J Obes (Lond). 2015 Oct 13;40(1):14–21. doi: 10.1038/ijo.2015.192 (PMC4722236; doi:10.1038/ijo.2015.192)
Supplement: Supplementary Table 3S [file ijo2015192x3.docx]

**Table 3S.** Unadjusted associations Between Visceral or Subcutaneous Abdominal Fat and Attained Weight and Height Z-Score in Different Ages from Birth to 30 Years, Stratified by Sex.

| Outcomes/ age | Weight (Z-score) | | | | |  | Height (Z-score) | | | | |
| --- | --- | --- | --- | --- | --- | --- | --- | --- | --- | --- | --- |
|  | N | β* | 95%CI | | p |  | N | β* | 95%CI | | p |
| ***Visceral fat thickness (SD ln cm)*** | | | | | | | | | | | |
| **Males** |  |  |  |  |  |  |  |  |  |  |  |
| Birth | 1724 | 0.02 | -0.01 | 0.06 | 0.19 |  |  |  |  |  |  |
| 1y | 437 | 0.07 | 0 | 0.14 | 0.05 |  | 437 | 0.03 | -0.03 | 0.1 | 0.34 |
| 2y | 1574 | 0.03 | 0 | 0.07 | 0.09 |  | 1574 | 0.01 | -0.02 | 0.05 | 0.38 |
| 4y | **1551** | **0.07** | **0.03** | **0.1** | **0.001** |  | 1550 | 0.03 | -0.01 | 0.07 | 0.11 |
| 15y | **384** | **0.18** | **0.1** | **0.26** | **<0.001** |  | **384** | **0.11** | **0.03** | **0.2** | **0.01** |
| 18/19y | **1570** | **0.25** | **0.21** | **0.28** | **<0.001** |  | 1571 | 0 | -0.04 | 0.04 | 0.99 |
| 23y | **1587** | **0.34** | **0.3** | **0.38** | **<0.001** |  | 1588 | 0 | -0.04 | 0.04 | 0.88 |
| 30y | **1721** | **0.49** | **0.46** | **0.52** | **<0.001** |  | 1721 | -0.01 | -0.05 | 0.03 | 0.62 |
| **Females** |  |  |  |  |  |  |  |  |  |  |  |
| Birth | 1768 | -0.02 | -0.05 | 0.02 | 0.38 |  |  |  |  |  |  |
| 1y | 499 | -0.07 | -0.15 | 0.01 | 0.07 |  | **499** | **-0.11** | **-0.18** | **-0.03** | **0.005** |
| 2y | 1628 | -0.03 | -0.07 | 0.02 | 0.2 |  | **1629** | **-0.07** | **-0.11** | **-0.03** | **0.001** |
| 4y | 1583 | 0 | -0.04 | 0.04 | 0.97 |  | **1583** | **-0.05** | **-0.09** | **-0.01** | **0.01** |
| 15y | **384** | **0.2** | **0.12** | **0.29** | **<0.001** |  | 384 | -0.05 | -0.15 | 0.04 | 0.24 |
| 18/19y^a^ | **686** | **0.2** | **0.14** | **0.27** | **<0.001** |  | 686 | -0.07 | -0.14 | 0 | 0.06 |
| 23y^b^ | **1502** | **0.36** | **0.32** | **0.41** | **<0.001** |  | **1610** | **-0.07** | **-0.11** | **-0.02** | **0.004** |
| 30y | **1765** | **0.52** | **0.48** | **0.56** | **<0.001** |  | **1765** | **-0.07** | **-0.12** | **-0.03** | **0.001** |
| ***Subcutaneous abdominal fat thickness (SD sqrt cm)*** | | | | | | | | | | | |
| **Males** |  |  |  |  |  |  |  |  |  |  |  |
| Birth | **1724** | **0.06** | **0.02** | **0.1** | **0.002** |  |  |  |  |  |  |
| 1y | **437** | **0.15** | **0.07** | **0.23** | **<0.001** |  | **437** | **0.11** | **0.04** | **0.19** | **0.004** |
| 2y | **1574** | **0.2** | **0.16** | **0.25** | **<0.001** |  | **1574** | **0.16** | **0.12** | **0.2** | **<0.001** |
| 4y | **1551** | **0.31** | **0.27** | **0.35** | **<0.001** |  | **1550** | **0.22** | **0.18** | **0.26** | **<0.001** |
| 15y | **384** | **0.47** | **0.4** | **0.55** | **<0.001** |  | **384** | **0.21** | **0.12** | **0.3** | **<0.001** |
| 18/19y | **1570** | **0.53** | **0.49** | **0.57** | **<0.001** |  | **1571** | **0.16** | **0.11** | **0.21** | **<0.001** |
| 23y | **1587** | **0.59** | **0.55** | **0.63** | **<0.001** |  | **1588** | **0.16** | **0.11** | **0.21** | **<0.001** |
| 30y | **1721** | **0.69** | **0.66** | **0.72** | **<0.001** |  | **1721** | **0.15** | **0.1** | **0.19** | **<0.001** |
| **Females** |  |  |  |  |  |  |  |  |  |  |  |
| Birth | 1768 | 0.04 | 0 | 0.08 | 0.07 |  |  |  |  |  |  |
| 1y | **499** | **0.12** | **0.04** | **0.2** | **0.003** |  | 499 | 0.06 | -0.01 | 0.14 | 0.1 |
| 2y | **1628** | **0.15** | **0.1** | **0.2** | **<0.001** |  | **1629** | **0.06** | **0.02** | **0.1** | **0.003** |
| 4y | **1583** | **0.23** | **0.18** | **0.27** | **<0.001** |  | **1583** | **0.09** | **0.05** | **0.14** | **<0.001** |
| 15y | **384** | **0.58** | **0.5** | **0.66** | **<0.001** |  | **384** | **0.16** | **0.06** | **0.25** | **0.002** |
| 18/19y^a^ | **686** | **0.48** | **0.42** | **0.54** | **<0.001** |  | **686** | **0.1** | **0.03** | **0.17** | **0.01** |
| 23y^b^ | **1502** | **0.62** | **0.58** | **0.65** | **<0.001** |  | 1610 | 0.04 | -0.01 | 0.09 | 0.12 |
| 30y | **1765** | **0.75** | **0.72** | **0.78** | **<0.001** |  | 1765 | 0.02 | -0.03 | 0.07 | 0.39 |

*Coefficients are from multiple linear regression and represent difference in the outcome per one standard deviation increase in the exposure.

^a^Excluding 27 pregnant women in 2000.

^b^Excluding 20 pregnant and 8 post-partum women in 2004–2005.
