# Supplementary material for: Associations of birth weight, linear growth and relative weight gain throughout life with abdominal fat depots in adulthood: the 1982 Pelotas (Brazil) birth cohort study
Source: Int J Obes (Lond). 2015 Oct 13;40(1):14–21. doi: 10.1038/ijo.2015.192 (PMC4722236; doi:10.1038/ijo.2015.192)
Supplement: Supplementary Table 4S [file ijo2015192x4.docx]

**Table 4S.** Unadjusted associations between visceral and subcutaneous abdominal fat thicknesses at age 30 years according to one unit s.d. increase in conditional relative weight gain or conditional height from birth to 30 years

| Sex/Age | **Conditional relative weight gain** | | | |  | **Conditional height** | | | |
| --- | --- | --- | --- | --- | --- | --- | --- | --- | --- |
|  | β | 95%CI | | p-value |  | β | 95%CI | | p-value |
| ***Visceral fat thickness (SD ln cm)*** | | | | | | | | | |
| **Males** | (N = 1,363) | | | |  | (N = 1,363) | | | |
| 2y | 0.03 | -0.02 | 0.07 | 0.21 |  |  |  |  |  |
| 4y | **0.07** | **0.03** | **0.12** | **0.002** |  | 0.02 | -0.03 | 0.06 | 0.49 |
| 23y | **0.37** | **0.34** | **0.41** | **<0.001** |  | -0.03 | -0.07 | 0.01 | 0.19 |
| 30y | **0.41** | **0.38** | **0.45** | **<0.001** |  |  |  |  |  |
| **Females^a,b^** | (N = 1,300) | | | |  | (N = 1,300) | | | |
| 2y | 0.03 | -0.02 | 0.08 | 0.2 |  |  |  |  |  |
| 4y | **0.08** | **0.03** | **0.14** | **0.001** |  | 0.02 | -0.03 | 0.07 | 0.47 |
| 23y | **0.41** | **0.37** | **0.46** | **<0.001** |  | -0.02 | -0.07 | 0.03 | 0.35 |
| 30y | **0.43** | **0.38** | **0.48** | **<0.001** |  |  |  |  |  |
| ***Subcutaneous abdominal fat thickness (SD sqrt cm)*** | | | | | | | | | |
| **Males** | (N = 1,363) | | | |  | (N = 1,363) | | | |
| 2y | **0.12** | **0.07** | **0.17** | **<0.001** |  |  |  |  |  |
| 4y | **0.22** | **0.17** | **0.27** | **<0.001** |  | **0.1** | **0.05** | **0.15** | **<0.001** |
| 23y | **0.5** | **0.46** | **0.54** | **<0.001** |  | 0.01 | -0.04 | 0.06 | 0.61 |
| 30y | **0.38** | **0.33** | **0.43** | **<0.001** |  |  |  |  |  |
| **Females^a,b^** | (N = 1,300) | | | |  | (N = 1,300) | | | |
| 2y | **0.15** | **0.1** | **0.2** | **<0.001** |  |  |  |  |  |
| 4y | **0.24** | **0.19** | **0.29** | **<0.001** |  | 0.03 | -0.02 | 0.08 | 0.25 |
| 23y | **0.58** | **0.54** | **0.63** | **<0.001** |  | -0.02 | -0.08 | 0.03 | 0.4 |
| 30y | **0.44** | **0.39** | **0.49** | **<0.001** |  |  |  |  |  |

*Using WAZ at birth according to the WHO growth curves.

^a^Excluding 27 pregnant women in 2000.

^b^Excluding 20 pregnant and 8 post-partum women in 2004–2005.
