# Supplementary material for: Associations of birth weight, linear growth and relative weight gain throughout life with abdominal fat depots in adulthood: the 1982 Pelotas (Brazil) birth cohort study
Source: Int J Obes (Lond). 2015 Oct 13;40(1):14–21. doi: 10.1038/ijo.2015.192 (PMC4722236; doi:10.1038/ijo.2015.192)
Supplement: Supplementary Table 5S [file ijo2015192x5.docx]

**Table 5S.** Coefficients From Multiple Linear Regression for Ultrasound Measurements of Abdominal Fat According to One Unit Standard Deviation Increase in Weight and Height Z-Score Changes in Different Periods From Birth to 30 Years. Stratified by Sex.

| Sex/Period | **Weight Z-score changes** | | | | | | | | | | |  | **Height Z-score changes** | | | | | | | | | | |
| --- | --- | --- | --- | --- | --- | --- | --- | --- | --- | --- | --- | --- | --- | --- | --- | --- | --- | --- | --- | --- | --- | --- | --- |
|  | Model 1 | | | | |  | Model 2 | | | | |  | Model 1 | | | | |  | Model 2 | | | | |
|  | N | β | 95%CI | | p-value |  | N | β | 95%CI | | p-value |  | N | β | 95%CI | | p-value |  | N | β | 95%CI | | p-value |
| ***Visceral fat thickness (SD ln cm)*** | | | | | | | | | | | | | | | | | | | | | | | |
| **Males** |  |  |  |  |  |  |  |  | | | |  |  |  |  |  |  |  |  |  | | | |
| 0 to 2 | 1363 | 0.03 | -0.01 | 0.07 | 0.18 |  | 1333 | 0.03 | -0.02 | 0.07 | 0.27 |  |  |  |  |  |  |  |  |  |  |  |  |
| 0 to 1 | 437 | 0.08 | 0.00 | 0.16 | 0.06 |  | 416 | 0.08 | 0.00 | 0.17 | 0.06 |  |  |  |  |  |  |  |  |  |  |  |  |
| 1 to 2 | 408 | -0.02 | -0.16 | 0.12 | 0.74 |  | 389 | -0.02 | -0.17 | 0.13 | 0.77 |  | 408 | 0.07 | -0.05 | 0.18 | 0.25 |  | 389 | 0.06 | -0.06 | 0.18 | 0.33 |
| 2 to 4 | **1469** | **0.11** | **0.03** | **0.18** | **0.004** |  | **1383** | **0.11** | **0.03** | **0.18** | **0.01** |  | 1468 | 0.04 | -0.02 | 0.11 | 0.21 |  | 1382 | 0.06 | -0.02 | 0.13 | 0.13 |
| 4 to 23 | **1363** | **0.43** | **0.39** | **0.48** | **<0.001** |  | **1333** | **0.45** | **0.40** | **0.49** | **<0.001** |  | 1363 | -0.05 | -0.11 | 0.02 | 0.15 |  | 1333 | -0.05 | -0.11 | 0.02 | 0.17 |
| 4 to 15 | **361** | **0.25** | **0.14** | **0.35** | **<0.001** |  | **341** | **0.23** | **0.12** | **0.34** | **<0.001** |  | **361** | **0.20** | **0.08** | **0.32** | **0.001** |  | **341** | **0.20** | **0.07** | **0.33** | **0.003** |
| 15 to 18/19 | **377** | **0.26** | **0.12** | **0.40** | **<0.001** |  | **357** | **0.27** | **0.12** | **0.42** | **<0.001** |  | 377 | -0.09 | -0.21 | 0.03 | 0.15 |  | 357 | -0.07 | -0.20 | 0.07 | 0.35 |
| 18/19 to 23 | **1473** | **0.53** | **0.46** | **0.61** | **<0.001** |  | **1403** | **0.55** | **0.47** | **0.63** | **<0.001** |  | 1475 | -0.06 | -0.24 | 0.12 | 0.49 |  | 1405 | -0.06 | -0.24 | 0.12 | 0.52 |
| 23 to 30 | **1584** | **0.80** | **0.73** | **0.86** | **<0.001** |  | **1506** | **0.79** | **0.73** | **0.85** | **<0.001** |  |  |  |  |  |  |  |  |  |  |  |  |
| **Females^a,b^** |  |  |  |  |  |  |  |  | | | |  |  |  |  |  |  |  |  |  |  |  |  |
| 0 to 2 | 1300 | -0.04 | -0.10 | 0.01 | 0.12 |  | 1276 | -0.01 | -0.07 | 0.05 | 0.78 |  |  |  |  |  |  |  |  |  |  |  |  |
| 0 to 1 | 498 | -0.04 | -0.13 | 0.05 | 0.43 |  | 450 | 0.01 | -0.09 | 0.11 | 0.90 |  |  |  |  |  |  |  |  |  |  |  |  |
| 1 to 2 | 471 | 0.02 | -0.15 | 0.19 | 0.83 |  | 426 | -0.02 | -0.21 | 0.17 | 0.82 |  | 471 | -0.09 | -0.21 | 0.03 | 0.15 |  | 426 | -0.08 | -0.21 | 0.05 | 0.24 |
| 2 to 4 | **1506** | **0.11** | **0.03** | **0.18** | **0.01** |  | **1377** | **0.10** | **0.02** | **0.18** | **0.02** |  | 1507 | 0.03 | -0.04 | 0.11 | 0.35 |  | 1377 | 0.05 | -0.03 | 0.12 | 0.22 |
| 4 to 23 | **1300** | **0.46** | **0.41** | **0.51** | **<0.001** |  | **1276** | **0.44** | **0.38** | **0.49** | **<0.001** |  | 1300 | -0.06 | -0.13 | 0.01 | 0.09 |  | **1276** | **-0.09** | **-0.16** | **-0.01** | **0.02** |
| 4 to 15 | **360** | **0.30** | **0.20** | **0.41** | **<0.001** |  | **330** | **0.28** | **0.16** | **0.39** | **<0.001** |  | 359 | 0.04 | -0.09 | 0.17 | 0.56 |  | 329 | 0.02 | -0.13 | 0.16 | 0.83 |
| 15 to 18/19 | **374** | **0.14** | **0.02** | **0.26** | **0.03** |  | 344 | 0.12 | 0.00 | 0.24 | 0.06 |  | 374 | -0.16 | -0.39 | 0.07 | 0.17 |  | 344 | -0.24 | -0.50 | 0.02 | 0.07 |
| 18/19 to 23 | **610** | **0.41** | **0.31** | **0.51** | **<0.001** |  | **558** | **0.40** | **0.30** | **0.50** | **<0.001** |  | 659 | -0.05 | -0.29 | 0.20 | 0.71 |  | 606 | 0.06 | -0.20 | 0.31 | 0.66 |
| 23 to 30 | **1499** | **0.83** | **0.76** | **0.90** | **<0.001** |  | **1384** | **0.85** | **0.77** | **0.92** | **<0.001** |  |  |  |  |  |  |  |  |  |  |  |  |
| ***Subcutaneous abdominal fat thickness (SD sqrt cm)*** | | | | | | | | | | | | | | | | | | | | | | | |
| **Males** |  |  |  |  |  |  |  |  | | | |  |  |  |  |  |  |  |  |  |  |  |  |
| 0 to 2 | **1363** | **0.21** | **0.16** | **0.26** | **<0.001** |  | **1333** | **0.17** | **0.12** | **0.22** | **<0.001** |  |  |  |  |  |  |  |  |  |  |  |  |
| 0 to 1 | **437** | **0.15** | **0.06** | **0.24** | **0.001** |  | **416** | **0.11** | **0.02** | **0.20** | **0.02** |  |  |  |  |  |  |  |  |  |  |  |  |
| 1 to 2 | **408** | **0.27** | **0.11** | **0.42** | **0.001** |  | **389** | **0.21** | **0.06** | **0.37** | **0.01** |  | **408** | **0.25** | **0.13** | **0.37** | **<0.001** |  | **389** | **0.23** | **0.10** | **0.36** | **<0.001** |
| 2 to 4 | **1469** | **0.42** | **0.34** | **0.50** | **<0.001** |  | **1383** | **0.40** | **0.32** | **0.48** | **<0.001** |  | **1468** | **0.20** | **0.12** | **0.28** | **<0.001** |  | **1382** | **0.18** | **0.10** | **0.26** | **<0.001** |
| 4 to 23 | **1363** | **0.60** | **0.55** | **0.64** | **<0.001** |  | **1333** | **0.59** | **0.54** | **0.64** | **<0.001** |  | 1363 | 0.00 | -0.07 | 0.07 | 0.92 |  | 1333 | 0.00 | -0.08 | 0.07 | 0.91 |
| 4 to 15 | **361** | **0.48** | **0.39** | **0.58** | **<0.001** |  | **341** | **0.46** | **0.36** | **0.56** | **<0.001** |  | 361 | 0.12 | -0.01 | 0.25 | 0.06 |  | 341 | 0.09 | -0.05 | 0.22 | 0.21 |
| 15 to 18/19 | **377** | **0.34** | **0.21** | **0.47** | **<0.001** |  | **357** | **0.35** | **0.22** | **0.49** | **<0.001** |  | 377 | 0.03 | -0.10 | 0.15 | 0.68 |  | 357 | 0.03 | -0.11 | 0.17 | 0.68 |
| 18/19 to 23 | **1473** | **0.53** | **0.45** | **0.61** | **<0.001** |  | **1403** | **0.53** | **0.45** | **0.61** | **<0.001** |  | 1475 | 0.00 | -0.21 | 0.21 | 0.98 |  | 1405 | -0.01 | -0.22 | 0.20 | 0.93 |
| 23 to 30 | **1584** | **0.75** | **0.68** | **0.81** | **<0.001** |  | **1506** | **0.76** | **0.69** | **0.82** | **<0.001** |  |  |  |  |  |  |  |  |  |  |  |  |
| **Females^a,b^** |  |  |  |  |  |  |  |  | | | |  |  |  |  |  |  |  |  |  |  |  |  |
| 0 to 2 | **1300** | **0.16** | **0.10** | **0.21** | **<0.001** |  | **1276** | **0.17** | **0.11** | **0.23** | **<0.001** |  |  |  |  |  |  |  |  |  |  |  |  |
| 0 to 1 | **498** | **0.15** | **0.06** | **0.24** | **0.001** |  | **450** | **0.13** | **0.03** | **0.22** | **0.01** |  |  |  |  |  |  |  |  |  |  |  |  |
| 1 to 2 | **471** | **0.34** | **0.17** | **0.52** | **<0.001** |  | **426** | **0.25** | **0.06** | **0.44** | **0.01** |  | 471 | 0.07 | -0.06 | 0.19 | 0.31 |  | 426 | -0.01 | -0.14 | 0.12 | 0.91 |
| 2 to 4 | **1506** | **0.36** | **0.28** | **0.44** | **<0.001** |  | **1377** | **0.33** | **0.25** | **0.42** | **<0.001** |  | **1507** | **0.13** | **0.06** | **0.21** | **0.001** |  | **1377** | **0.14** | **0.06** | **0.22** | **<0.001** |
| 4 to 23 | **1300** | **0.65** | **0.60** | **0.69** | **<0.001** |  | **1276** | **0.64** | **0.59** | **0.69** | **<0.001** |  | **1300** | **-0.09** | **-0.17** | **-0.02** | **0.02** |  | **1276** | **-0.11** | **-0.19** | **-0.03** | **0.01** |
| 4 to 15 | **360** | **0.64** | **0.54** | **0.73** | **<0.001** |  | **330** | **0.62** | **0.52** | **0.73** | **<0.001** |  | 359 | 0.12 | -0.02 | 0.26 | 0.10 |  | 329 | 0.06 | -0.09 | 0.22 | 0.43 |
| 15 to 18/19 | **374** | **0.16** | **0.05** | **0.26** | **0.004** |  | **344** | **0.16** | **0.05** | **0.27** | **0.004** |  | **374** | **-0.31** | **-0.56** | **-0.06** | **0.01** |  | 344 | -0.26 | -0.53 | 0.01 | 0.06 |
| 18/19 to 23 | **610** | **0.59** | **0.50** | **0.68** | **<0.001** |  | **558** | **0.62** | **0.52** | **0.71** | **<0.001** |  | 659 | 0.06 | -0.20 | 0.33 | 0.65 |  | 606 | 0.14 | -0.14 | 0.41 | 0.33 |
| 23 to 30 | **1499** | **0.87** | **0.81** | **0.93** | **<0.001** |  | **1384** | **0.86** | **0.79** | **0.92** | **<0.001** |  |  |  |  |  |  |  |  |  |  |  |  |

Model 1 - Adjusted for height z-score at the beginning of the period. Model 2 - Adjusted as model 1 + confounders measured at the time of birth: family income at birth, maternal education, maternal skin colour, maternal height, maternal BMI before the pregnancy (imputed), smoking in pregnancy, and gestational age (imputed). ^a^Excluding 27 pregnant women in 2000. ^b^Excluding 20 pregnant and 8 post-partum women in 2004–2005.
