# Supplementary material for: Associations of birth weight, linear growth and relative weight gain throughout life with abdominal fat depots in adulthood: the 1982 Pelotas (Brazil) birth cohort study
Source: Int J Obes (Lond). 2015 Oct 13;40(1):14–21. doi: 10.1038/ijo.2015.192 (PMC4722236; doi:10.1038/ijo.2015.192)
Supplement: Supplementary Table 6S [file ijo2015192x6.docx]

**Table 6S.** Means and Standard Deviations (s.d.) of Visceral and Subcutaneous Abdominal Fat Thickness According to Intrauterine Growth Restriction and Nutritional Status at Age 2 Years

| Variables | N | Visceral fat thickness  (SD ln cm) | | |  | Subcutaneous abdominal fat thickness  (SD sqrt cm) | | |
| --- | --- | --- | --- | --- | --- | --- | --- | --- |
|  |  | Mean | s.d. | p-value |  | Mean | s.d. | p-value |
| **Males** |  |  |  |  |  |  |  |  |
| IUGR |  |  |  |  |  |  |  |  |
| Not IUGR | 1144 | 0.51 | 0.82 | 0.35 |  | -0.25 | 0.95 | **<0.001** |
| IUGR | 212 | 0.45 | 0.73 |  |  | -0.51 | 0.90 |  |
| Stunting at 2y |  |  |  |  |  |  |  |  |
| No | 1169 | 0.50 | 0.81 | 0.85 |  | -0.23 | 0.94 | **<0.001** |
| Yes | 194 | 0.49 | 0.80 |  |  | -0.63 | 0.90 |  |
| Overweight at 2y |  |  |  |  |  |  |  |  |
| No | 1235 | 0.49 | 0.80 | 0.11 |  | -0.33 | 0.93 | **<0.001** |
| Yes | 128 | 0.61 | 0.84 |  |  | 0.08 | 0.95 |  |
| **Females^a,b^** |  |  |  |  |  |  |  |  |
| IUGR |  |  |  |  |  |  |  |  |
| Not IUGR | 1086 | -0.55 | 0.93 | **0.01** |  | 0.29 | 0.97 | 0.33 |
| IUGR | 206 | -0.37 | 0.82 |  |  | 0.36 | 0.98 |  |
| Stunting at 2y |  |  |  |  |  |  |  |  |
| No | 1159 | -0.53 | 0.91 | 0.25 |  | 0.32 | 0.97 | **0.03** |
| Yes | 141 | -0.44 | 0.95 |  |  | 0.14 | 0.95 |  |
| Overweight at 2y |  |  |  |  |  |  |  |  |
| No | 1197 | -0.53 | 0.91 | 0.43 |  | 0.27 | 0.96 | **<0.001** |
| Yes | 103 | -0.45 | 0.97 |  |  | 0.69 | 0.99 |  |

Abbreviation: IUGR, intrauterine growth restriction.

^a^Excluding 27 pregnant women in 2000.

^b^Excluding 20 pregnant and 8 post-partum women in 2004–2005.
